# Acupuncture for the Treatment of Alzheimer's Disease: An Overview of Systematic Reviews

**DOI:** 10.3389/fnagi.2020.574023

**Published:** 2020-11-27

**Authors:** Jinke Huang, Min Shen, Xiaohui Qin, Manli Wu, Simin Liang, Yong Huang

**Affiliations:** ^1^The Second Clinical Medical College of Guangzhou University of Chinese Medicine, Guangzhou, China; ^2^Department of Neurology, Guangdong Provincial Hospital of Chinese Medicine, Guangzhou, China; ^3^School of Traditional Chinese Medicine, Southern Medical University, Guangzhou, China

**Keywords:** acupuncture, Alzheimer's disease, overview, systematic reviews, treatment

## Abstract

**Background:** Acupuncture may be an effective complementary treatment for Alzheimer's disease (AD). The aim of this study was to summarize the evidence provided by systematic reviews (SRs)/meta-analyses (MAs) on the effect of acupuncture on AD.

**Methods:** Eight electronic databases were searched from their inception until October 19, 2020. The methodological quality, reporting quality, and risk of bias of the included SRs were assessed by the Assessing the Methodological Quality of Systematic Reviews 2 (AMSTAR-2), the Risk of Bias in Systematic Reviews (ROBIS) tool, and the Preferred Reporting Items for Systematic Reviews and Meta-Analyses (PRISMA). Moreover, the evidence quality of the outcome measures was assessed by the Grading of Recommendations, Assessment, Development, and Evaluation (GRADE).

**Results:** Eleven SRs/MAs met all inclusion criteria. According to the results of the AMSTAR-2, all included reviews were rated critically as being of low quality. With PRISMA, the reporting checklist was relatively complete, but some reporting weaknesses remained in the topics of the protocol and registration, search strategy, risk of bias, additional analyses, and funding. Based on the ROBIS tool, only two SRs/MAs had a low risk of bias. With the GRADE system, no high-quality evidence was found, and only seven outcomes provided moderate-quality evidence. Among the downgraded factors, the risk of bias within the original trials was ranked first, followed by inconsistency, imprecision, and publication bias.

**Conclusions:** Acupuncture is a promising complementary treatment for AD. However, due to the low quality of the SRs/MAs supporting these results, high-quality studies with rigorous study designs and larger samples are needed before widespread recommendations can be made.

## Introduction

Alzheimer's disease (AD) is a common progressive degenerative encephalopathy characterized by cognitive impairment, declining memory, emotional changes, and a language barrier (McKhann et al., 1984). AD seriously affects the physical health and quality of life of patients and places a heavy burden on families and society (Xu et al., 2017). With the extension of the average life expectancy of the population, the incidence of AD is increasing annually; the number of patients worldwide is currently as high as 24 million, which is expected to increase 4-fold by 2050 (Reitz and Mayeux, 2014). Currently, no medication can prevent, halt, or reverse the progression of AD. The clinical drugs approved by the Food and Drug Administration (FDA) for AD only have modest symptomatic effects (Atri et al., 2008) and have been related to many adverse reactions (Tampi and Dyck, 2007). Hence, some patients choose complementary and alternative medicine to treat AD in an effort to improve their quality of life. Worldwide, acupuncture has been accepted as a popular and safe complementary therapy (Bodeker et al., 2005), and it has been widely used to treat AD by physicians aiming to reduce the side effects of medication and to increase its therapeutic effectiveness.

Systematic reviews (SRs)/meta-analyses (MAs) are important tools to guide evidence-based clinical practice, and they have been widely used in various medical fields in recent years. However, with the increasing number of SRs/MAs, their quality is uneven, and their conclusions on the same topic of SR/MAs are often contradictory; therefore, the clinical evidence they provide has been criticized. A systematic overview of SRs/MAs is a relatively new approach for synthesizing the outcomes from multiple SRs/MAs, evaluating their quality and attempting to address any inconsistent outcomes. The objective of our study was to critically assess the scientific quality of relevant SRs/MAs regarding the application of acupuncture in the treatment of PD using a systematic overview.

## Materials and Methods

### Eligibility Criteria

#### Type of Studies

This study included SRs/MAs of randomized controlled trials (RCTs) of patients who were diagnosed with AD using definitive diagnostic criteria. Repeated publications, graduate dissertations, and SRs/MAs that were not rigorous were excluded.

#### Interventions

Studies of acupuncture (e.g., manual acupuncture, auricular acupuncture, or needling) or acupuncture plus conventional therapy (CT) as an intervention for AD were included. The following treatments were used in the control group: medication, placebo, and no treatment. The control group treatments were CT alone or placebo.

#### Outcome Indicators

SRs/MAs should have at least one clear outcome such as the effective rate, Ability of Daily Living (ADL), Mini-Mental State Examination (MMSE), Alzheimer's Disease Assessment Scale-Cognition (ADAS-cog), Hasegawa's Dementia Scale (HDS), mood, or behavior.

### Data Sources and Search Strategy

Eight electronic databases [Web of Science, The Cochrane Library, PubMed, EMBASE, Sino-Med, China National Knowledge Infrastructure (CNKI), Wanfang Database, and Chongqing VIP] from their inception until February 21, 2020, were searched for potential SRs/MAs, and we conducted an updated search on October 19, 2020, to provide more up-to-date and comprehensive evidence. The search strategies for each database are presented in Appendix 1.

### Data Management and Extraction

All articles were read by two independent investigators, and data from the articles were validated and extracted according to the predefined criteria. Disagreements between the two investigators were resolved through discussion.

### Quality Assessment

Two independent investigators assessed the methodological quality, reporting quality, risk of bias, and evidence quality by the Assessing the Methodological Quality of Systematic Reviews 2 (AMSTAR-2) (Shea et al., 2017), Preferred Reporting Items for Systematic Reviews and Meta-Analyses (PRISMA) for the acupuncture checklist (Wang et al., 2019a), Risk of Bias in Systematic Reviews (ROBIS) (Whiting et al., 2016), and Grading of Recommendations, Assessment, Development, and Evaluation (GRADE) (Atkins et al., 2004), respectively. Disagreements between the two investigators were resolved through discussion.

The AMSTAR-2 is a valid instrument composed of 16 items, and each item could be evaluated as “yes,” “partial yes,” or “no.” After interpreting the weaknesses detected in all items, the overall rating of the quality can be rated as “high,” “moderate,” “low,” or “critically low” (Shea et al., 2017). The PRISMA statement is a valid instrument composed of 27 items. Each item was evaluated as “yes,” “partial yes,” and “no,” representing full reports, partial reports, and no reports (Moher et al., 2010). The completion of each item is presented as a ratio. The ROBIS is a valid tool composed of three phases for evaluating the level of bias present within an SR. The risk of bias can be rated as “low,” “high,” or “unclear” (Whiting et al., 2016). The GRADE system assesses evidence quality with four levels: high, moderate, low, or very low. The initial grading would be decreased if there were study limitations, inconsistencies, imprecision, indirectness, or publication bias (Atkins et al., 2004).

## Results

### Results on Literature Search and Selection

**Figure 1:**
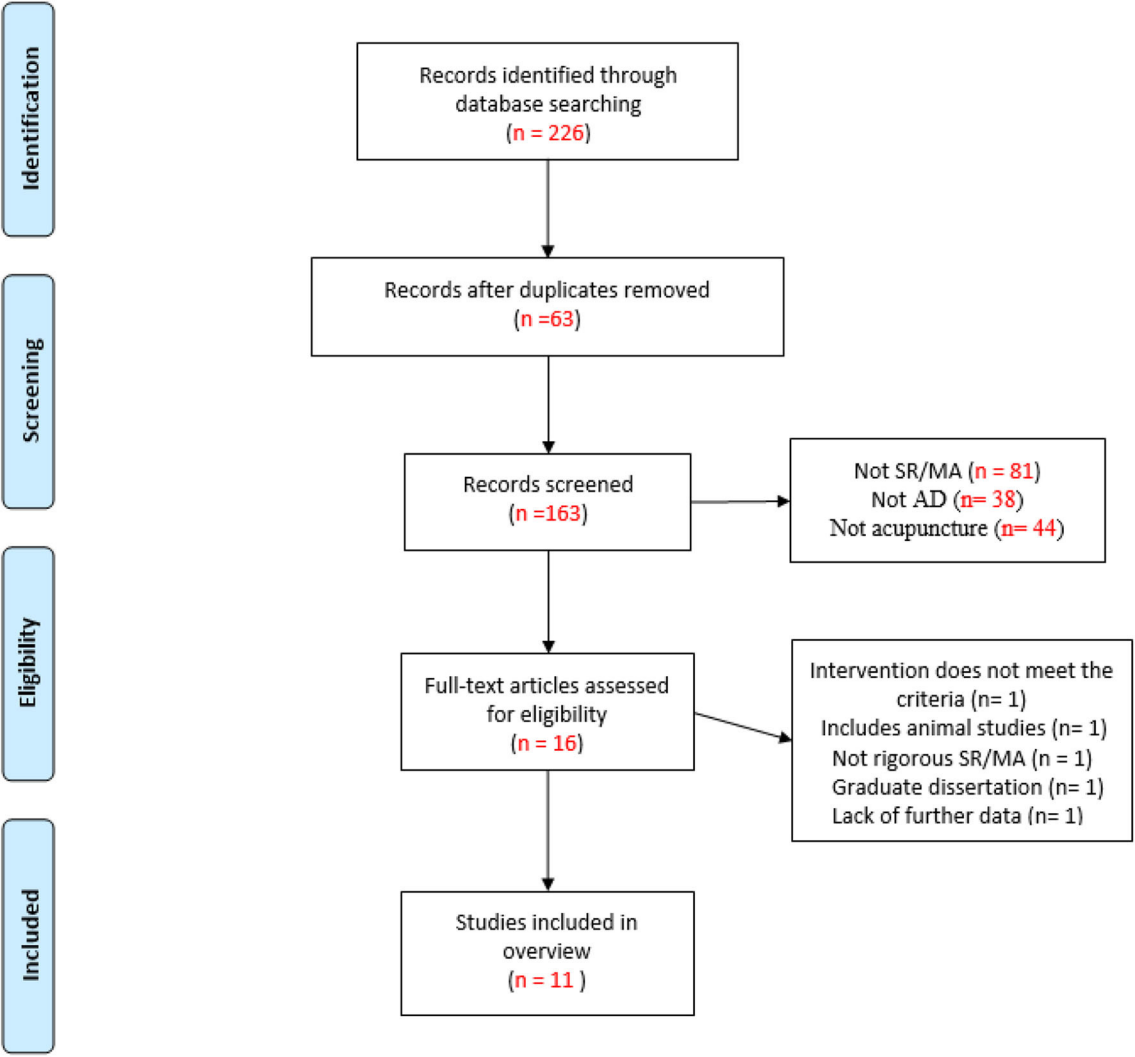
The flowchart of the literature selection.

In total, 226 publications were retrieved from the eight databases. After removing duplicates and title/abstract screening, 16 publications were retrieved for full-text assessment. Examining these full-text publications resulted in the exclusion of four publications (Appendix 2). Finally, 11 publications (Guo et al., 2008; Lee et al., 2009; Cao et al., 2014; Xu and Xie, 2015; Zhou et al., 2015, 2017; Zou et al., 2016; Lin et al., 2017; Huang et al., 2019; Wang et al., 2019b, 2020) were selected for inclusion in this overview. The screening and selection procedure is presented in Figure 1.

### Description of Characteristics

The summarized data extracted from the 11 SRs/MAs are presented in Table 1. These included SRs/MAs that were published in the period from 2008 to 2020. Six of them were written in Chinese (Guo et al., 2008; Cao et al., 2014; Xu and Xie, 2015; Zou et al., 2016; Lin et al., 2017; Wang et al., 2019b), and the remaining five (Lee et al., 2009; Zhou et al., 2015, 2017; Huang et al., 2019; Wang et al., 2020) were written in English. These SRs/MAs were all published by authors from East Asia (10 from China and one from Korea). The number of trials ranged between three and 31, and the sample size ranged from 166 to 2,045. Interventions in the therapy group were mainly acupuncture or acupuncture combined with CT, while CT or sham acupuncture was used in the control group. In terms of the quality assessment scales, two (Guo et al., 2008; Xu and Xie, 2015) used Jadad, and the others used the Cochrane risk of bias criteria. Three (one was published in English and two were published in Chinese) of the 11 included SRs/MAs reached negative conclusions, and the remaining eight reached positive conclusions (four were published in English and four were published in Chinese).

**Table 1:** Main characteristics of the included reviews.

**Author, year (country)**	**Language**	**Trials (subjects)**	**Treatment intervention**	**Control intervention**	**Quality assessment**	**Meta-analyses**	**Results**	**Results summary**
Wang et al. (2019b) (China)	Chinese	8 (472)	CA + CT	CT	Cochrane criteria	Yes	Acupuncture combined with medicine for cognitive functions and life quality of AD patients is effective	Positive
Lin et al. (2017) (China)	Chinese	13 (730)	CA, EA, AT + CT	CT	Cochrane criteria	Yes	Acupuncture treatment can improve the learning and memory ability of patients with AD	Positive
Zou et al. (2016) (China)	Chinese	8 (349)	CA, EA	CT	Cochrane criteria	Yes	The advantages of acupuncture in treating AD compared with medication are unsure	Negative
Xu and Xie (2015) (China)	Chinese	10 (652)	CA, EA, SA, CA + CT	CT	Jadad	Yes	Acupuncture combined with medication in AD treatment is definitely effective	Positive
Cao et al. (2014) (China)	Chinese	5 (233)	CA	CT	Cochrane criteria	Yes	Compared with medication, the acupuncture cannot improve the MMSE and ADL score in patients with AD	Negative
Guo et al. (2008) (China)	Chinese	22 (1,368)	EA, SA, CA	CT	Jadad	Yes	Acupuncture is effective on AD according to the domestic clinical literatures	Positive
Wang et al. (2020) (China)	English	31 (2,045)	EA, CA + CT	CT, sham acupuncture, no treatment	Cochrane criteria	Yes	Acupuncture plus drug therapy may have a more beneficial effect for AD patients than drug therapy alone on general cognitive function in the short term and medium term and on ADL skills in the medium term	Positive
Huang et al. (2019) (China)	English	13 (777)	CA, EA	CT	Cochranecriteria	Yes	Acupuncture alone is superior to CT for AD in most of the studies assessed in the current MAs	Positive
Zhou et al. (2017) (China)	English	15 (1,217)	CA + CT	CT	Cochrane criteria	Yes	From the current results, acupuncture plus medicine may have advantages over CT for treating AD	Positive
Zhou et al. (2015) (China)	English	10 (585)	SA, EA, CA + CT	CT; no treatment	Cochrane criteria	Yes	Acupuncture may enhance the effect of CT for treating AD in terms of improving cognitive function. Acupuncture may also be more effective than CT at improving AD patients' ability to carry out their daily lives	Positive
Lee et al. (2009) (Korea)	English	3 (166)	EA + CT	CT	Cochrane criteria	Yes	The existing evidence does not demonstrate the effectiveness of acupuncture for AD	Negative

*CA, conventional acupuncture; EA, electroacupuncture; SA, scalp acupuncture; EA, eye acupuncture; CT, conventional therapy*.

### Results of the Methodological Quality

Considering the methodological quality, all SRs/MAs were regarded as critically low quality because there was more than one critical item that was unmet in the included SRs/MAs. The methodological limitations arose from the following items: item 2 (only one SR/MA registered the protocol), item 4 (only three SRs/MAs provided a complete search strategy), item 7 (none of the SRs/MAs provided a list of excluded studies), item 13 [three SRs/MAs did not account for risk of bias (RoB) in the primary studies when interpreting the results of the review], and item 15 (four SRs/MAs did not conduct a publication bias study or discuss its impact on the review). The details are given in Table 2.

**Table 2:** Results of the AMSTAR-2 assessments.

**References**	**AMSTAR-2**																**Quality**
	**Q1**	**Q2**	**Q3**	**Q4**	**Q5**	**Q6**	**Q7**	**Q8**	**Q9**	**Q10**	**Q11**	**Q12**	**Q13**	**Q14**	**Q15**	**Q16**	
Wang et al. (2019b)	Y	PY	Y	PY	Y	Y	N	Y	Y	Y	Y	Y	Y	Y	Y	Y	CL
Lin et al. (2017)	Y	PY	Y	PY	Y	Y	N	Y	Y	Y	Y	Y	Y	Y	N	Y	CL
Zou et al. (2016)	Y	PY	Y	PY	Y	Y	N	Y	Y	N	Y	N	N	Y	N	N	CL
Xu and Xie (2015)	Y	PY	Y	PY	N	N	N	Y	Y	Y	Y	N	Y	Y	Y	Y	CL
Cao et al. (2014)	Y	PY	Y	PY	Y	Y	N	Y	Y	N	Y	Y	Y	Y	N	N	CL
Guo et al. (2008)	Y	PY	Y	PY	Y	Y	N	Y	Y	Y	Y	Y	Y	Y	Y	Y	CL
Wang et al. (2020)	Y	PY	Y	Y	Y	Y	N	Y	Y	Y	Y	Y	Y	Y	Y	Y	CL
Huang et al. (2019)	Y	PY	Y	Y	Y	Y	N	Y	Y	Y	Y	Y	Y	Y	Y	Y	CL
Zhou et al. (2017)	Y	PY	Y	PY	Y	Y	N	Y	Y	N	Y	Y	N	Y	Y	N	CL
Zhou et al. (2015)	Y	Y	Y	Y	Y	Y	N	Y	Y	Y	Y	Y	N	Y	Y	Y	CL
Lee et al. (2009)	Y	PY	Y	PY	Y	Y	N	Y	Y	Y	Y	Y	Y	Y	N	Y	CL

*Y, yes; PY, partial yes; N, no; CL, critically low; L, low; H, high*.

### Results of the Reporting Quality

Table 3 presents the overview of the PRISMA for the acupuncture checklist. In general, no SR/MA reported all items of the PRISMA, but both of them were adequately reported, over 60%. The results showed that more than half of the items were reported in 100% of all SRs/MAs, but there were still some reporting flaws in other items. In the section of the methods, the topic of the protocol and registration, diagnostic criteria in traditional medicine, search, risk of bias across studies, and additional analyses were reported inadequately (≤50%); in the section of the results, no SR/MA reported details of the “de-qi,” and the risk of bias and additional analyses were reported in only 54.5%; in the section of the discussion, limitations were reported in only 81.8%; in section of the funding, funding was reported in only 72.7%. More details are presented in Table 3.

**Table 3:** Results of the PRISMA for the acupuncture checklist.

**Section/topic**	**Items**	**Wang et al. (2019b)**	**Lin et al. (2017)**	**Zou et al. (2016)**	**Xu and Xie (2015)**	**Cao et al. (2014)**	**Guo et al. (2008)**	**Wang et al. (2019b)**	**Huang et al. (2019)**	**Zhou et al. (2017)**	**Zhou et al. (2015)**	**Lee et al. (2009)**	**Compliance (%)**
Title	Q1. Title	Y	Y	Y	Y	Y	Y	Y	Y	Y	Y	Y	100
Abstract	Q2. Structured summary	Y	Y	Y	Y	Y	Y	Y	Y	Y	Y	Y	100
Introduction	Q3. Rationale	Y	Y	Y	Y	Y	Y	Y	Y	Y	Y	Y	100
	Q4. Objectives	Y	Y	Y	Y	Y	Y	Y	Y	Y	Y	Y	100
Methods	Q5. Protocol and registration	N	N	N	N	N	N	N	N	N	Y	N	9.1
	Q6. Eligibility criteria	Y	Y	Y	Y	Y	Y	Y	Y	Y	Y	Y	100
	Q6a.1. Diagnostic criteria in Western medicine	Y	Y	Y	Y	Y	Y	Y	Y	Y	Y	N	100
	Q6a.2. Diagnostic criteria in traditional medicine	Y	N	Y	N	N	Y	N	N	Y	N	N	36.4
	Q6b. Types of acupuncture	N	Y	Y	Y	N	Y	Y	Y	N	Y	Y	63.6
	Q6c. Report measures for therapeutic effects	Y	Y	Y	Y	Y	Y	Y	Y	Y	Y	Y	100
	Q7. Information sources	Y	Y	Y	Y	Y	Y	Y	Y	Y	Y	Y	100
	Q8. Search	PY	PY	PY	PY	PY	PY	Y	Y	PY	Y	PY	27.3
	Q9. Study selection	Y	Y	Y	Y	N	Y	Y	Y	Y	Y	Y	90.9
	Q10. Data collection process	Y	Y	Y	Y	N	N	Y	Y	Y	Y	Y	81.8
	Q11. Data items	Y	Y	Y	Y	Y	Y	Y	Y	Y	Y	Y	100
	Q12. Risk of bias in individual studies	Y	Y	Y	Y	Y	Y	Y	Y	Y	Y	Y	100
	Q13. Summary measures	Y	Y	Y	Y	Y	Y	Y	Y	Y	Y	Y	100
	Q14. Synthesis of results	Y	Y	Y	Y	Y	Y	Y	Y	Y	Y	Y	100
	Q15. Risk of bias across studies	Y	N	N	Y	N	Y	Y	Y	Y	N	N	54.5
	Q16. Additional analyses	Y	N	Y	N	N	N	Y	Y	Y	Y	N	54.5
Results	Q17. Study selection	Y	Y	Y	Y	Y	Y	Y	Y	Y	Y	Y	100
	Q18. Study characteristics	Y	Y	Y	Y	Y	Y	Y	Y	Y	Y	Y	100
	Q18a. Describe details of “de-qi”	N	N	N	N	N	N	N	N	N	N	N	0
	Q19. Risk of bias within studies	Y	Y	Y	Y	Y	Y	Y	Y	Y	Y	Y	100
	Q20. Results of individual studies	Y	Y	Y	Y	Y	Y	Y	Y	Y	Y	Y	100
	Q21. Synthesis of results	Y	Y	Y	Y	Y	Y	Y	Y	Y	Y	Y	100
	Q22. Risk of bias across studies	N	N	Y	Y	N	Y	Y	Y	Y	N	N	54.5
	Q23. Additional analysis	N	N	Y	Y	N	N	Y	Y	Y	Y	N	54.5
Discussion	Q24. Summary of evidence	Y	Y	Y	Y	Y	Y	Y	Y	Y	Y	Y	100
	Q25. Limitations	Y	Y	N	Y	N	Y	Y	Y	Y	Y	Y	81.8
	Q26. Conclusions	Y	Y	Y	Y	Y	Y	Y	Y	Y	Y	Y	100
Funding	Q27. Funding	Y	Y	N	Y	N	Y	Y	Y	N	Y	Y	72.7

### Results of ROBIS Evaluation

For ROBIS, phase 1 assesses the relevance of the research topic, and all SRs/MAs were rated as having a low risk of bias. Domain 1 assessed the study eligibility criteria, and all SRs/MAs were rated at a low risk of bias. Domain 2 assessed the identification and selection studies, and 10 SRs/MAs had a low risk of bias. Domain 3 assessed the collection and study appraisal, and 10 SRs/MAs were at a low risk of bias. Domain 4 assessed the synthesis and findings, and 4 out of 11 SRs/MAs were rated as having a low risk of bias. Phase 3 considered the overall risk of bias in the reviews, and three SRs/MAs were at a low risk of bias. More details are presented in Table 4.

**Table 4:** Results of the ROBIS assessments.

**Reviews**	**Phase 1**	**Phase 2**				**Phase 3**
	**Assessing relevance**	**Domain 1: study eligibility criteria**	**Domain 2: identification and selection of studies**	**Domain 3: collection and study appraisal**	**Domain 4: synthesis and findings**	**Risk of bias in the review**
Wang et al. (2019b)	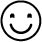	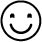	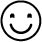	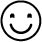	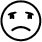	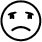
Lin et al. (2017)	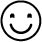	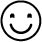	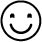	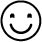	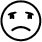	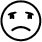
Zou et al. (2016)	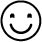	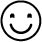	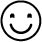	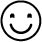	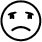	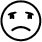
Xu and Xie (2015)	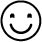	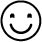	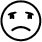	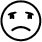	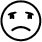	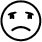
Cao et al. (2014)	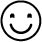	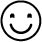	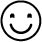	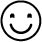	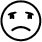	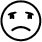
Guo et al. (2008)	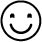	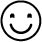	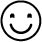	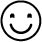	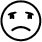	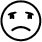
Wang et al. (2020)	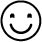	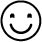	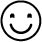	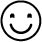	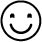	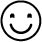
Huang et al. (2019)	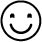	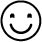	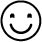	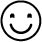	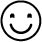	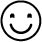
Zhou et al. (2017)	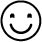	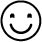	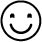	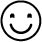	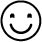	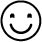
Zhou et al. (2015)	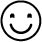	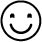	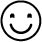	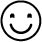	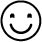	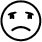
Lee et al. (2009)	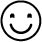	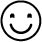	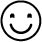	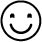	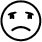	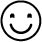

*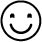, low risk; 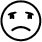, high risk*.

### Evidence Quality

Thirty-three outcomes were evaluated by the GRADE system. According to the evaluation results, no high-quality evidence was found, and only seven outcomes provided moderate-quality evidence. The evidence was downgraded due to limitations within the RCTs, inconsistency, imprecision, and publication bias. The details are given in Table 5.

**Table 5:** Results of evidence quality.

**Reviews**	**Outcomes**	**Limitations**	**Inconsistency**	**Indirectness**	**Imprecision**	**Publication bias**	**Relative effect (95% CI)**	***P-*value**	**Quality**
Wang et al. (2019b)	MMSE score	−1	−1	0	0	0	MD 0.76 (0.42, 1.10)	<0.0001	L
	ADAS-cog score	−1	0	0	−1	−1	MD −0.32 (−0.61, −0.03)	0.03	CL
	ADL score	−1	−1	0	−1	−1	MD −0.66 (−1.06, −0.27)	0.001	CL
Lin et al. (2017)	Effective rate	−1	−1	0	0	0	RR 1.16 (1.03, 1.31)	0.01	L
	MMSE score	−1	−1	0	−1	0	MD −0.99 (−3.45, 1.46)	>0.01	CL
Zou et al. (2016)	Effective rate	−1	0	0	−1	0	OR 1.15 (0.69, 1.91)	0.60	L
	MMSE score	−1	−1	0	0	0	MD 0.40 (−2.18, 2.97)	0.78	L
	ADL score	−1	−1	0	−1	0	MD 0.60 (−0.54, 1.74)	0.30	CL
	HDL score	−1	−1	0	−1	0	MD −0.20 (−1.19, 0.80)	0.70	CL
Xu and Xie (2015)	Effective rate	−1	0	0	0	0	RR 1.25 (1.14, 1.38)	<0.01	M
	MMSE score	−1	−1	0	−1	−1	MD 2.87 (0.64, 5.10)	0.01	CL
Cao et al. (2014)	MMSE score	−1	−1	0	−1	−1	WMD −0.61 (−1.34, 0.13)	0.11	CL
	ADL score	−1	−1	0	−1	−1	WMD −0.48 (−1.72, 0.76)	0.45	CL
Guo et al. (2008)	Effective rate	−1	0	0	0	0	OR 3.72 (2.73, 5.07)	<0.0001	M
Wang et al. (2020)	MMSE score	0	−1	0	0	0	MD 0.83 (0.14, 1.52)	0.02	M
	ADAS-cog score	0	−1	0	−1	0	MD −3.21 (−5.53, −0.89)	<0.01	L
	HDS score	0	0	0	−1	0	MD 0.58 (0.18, 0.99)	<0.01	M
	ADL score	0	0	0	−1	0	MD 0.21 (−0.74, 1.16)	0.66	M
Huang et al. (2019)	Effective rate	−1	0	0	0	0	RR 1.17 (1.06, 1.29)	0.001	M
	MMAE score	−1	−1	0	0	0	MD 1.96 (0.66, 3.26)	0.003	L
	ADAS-cog score	−1	−1	−1	0	−1	MD 3.56 (1.10, 6.03)	0.005	CL
	HDS score	−1	−1	0	0	0	MD −0.17 (−0.26, 0.90)	0.728	L
	ADL score	−1	−1	0	0	0	MD 1.99 (0.65, 3.34)	0.004	L
Zhou et al. (2017)	Effective rate	−1	0	0	0	0	OR 2.72 (2.04, 3.62)	<0.0001	M
	MMSE score	−1	−1	0	0	0	MD 2.10 (0.69, 3.51)	0.004	L
	ADL score	−1	−1	0	−1	−1	MD −3.59 (−7.18, 0.01)	0.05	CL
Zhou et al. (2015)	MMSE score	−1	−1	0	0	0	MD 1.05 (0.16, 1.93)	0.02	L
	HDS score	−1	0	0	−1	−1	SMD 0.09 (−0.28, 0.46)	0.62	CL
	ADL score	−1	0	0	−1	0	MD −2.80 (−4.57, −1.02)	0.002	L
	MMSE score	−1	0	0	−1	−1	MD 2.37 (1.53, 3.21)	<0.0001	CL
	ADL score	−1	0	0	−1	−1	MD −2.64 (−4.95, 0.32)	0.03	CL
Lee et al. (2009)	MMSE score	−1	0	0	−1	−1	MD −0.55 (−1.31, 0.21)	0.15	CL
	ADL score	−1	0	0	−1	−1	MD −1.29 (−1.77, −0.80)	<0.0001	CL

*−1, downgrade; 0, not downgrade; CL, critically low; L, low; M, moderate; H, high; MMSE, Mini-Mental State Examination Score; ADL, Activities of Daily Living Scale; ADAS-cog, Alzheimer's Disease Assessment Scale-Cognition; HDS, Hasegawa's Dementia Scale; MD, mean difference; RR, relative risk/risk ratio; OR, odds ratio; WMD, weighted mean difference; SMD, standardized mean difference*.

### Efficacy Evaluation

#### Acupuncture vs. CT

Eight studies (Guo et al., 2008; Lee et al., 2009; Cao et al., 2014; Zhou et al., 2015; Zou et al., 2016; Lin et al., 2017; Huang et al., 2019; Wang et al., 2020) compared the effects of acupuncture with CT. The effective rate of acupuncture in the treatment of AD was reported in four of seven SRs/MAs (Guo et al., 2008; Zou et al., 2016; Lin et al., 2017; Huang et al., 2019), and the results showed that acupuncture was superior to CT. Four SRs/MAs (Zhou et al., 2015; Zou et al., 2016; Lin et al., 2017; Huang et al., 2019) found significantly greater reductions in MMSE scores in the acupuncture group than in the CT group; however, there was no significant difference in the other three reviews (Lee et al., 2009; Cao et al., 2014; Wang et al., 2020). One SR/MA (Lee et al., 2009) revealed a significantly greater reduction in ADL scores in the acupuncture group than in the CT group, while there was no significant difference in the other five reviews (Cao et al., 2014; Zhou et al., 2015; Zou et al., 2016; Huang et al., 2019; Wang et al., 2020). Three SRs/MAs (Zhou et al., 2015; Huang et al., 2019; Wang et al., 2020) revealed that the HDL score and ADAS-cog score were significantly lower in the acupuncture group than in the CT group. For the HDS score, three SRs/MAs (Zou et al., 2016; Huang et al., 2019; Wang et al., 2020) revealed a significant decrease in the acupuncture group compared with the CT group.

#### Acupuncture Plus CT vs. CT

Four SRs/MAs (Xu and Xie, 2015; Zhou et al., 2015, 2017; Wang et al., 2019b) compared the effects of acupuncture plus medication with medication. Two out of the four SRs/MAs (Xu and Xie, 2015; Zhou et al., 2017) reported the effective rate of acupuncture plus CT for AD, and the results indicated that the combined treatment was superior to CT alone. The MMSE score was used to evaluate the efficacy of acupuncture for AD in four SRs/MAs (Xu and Xie, 2015; Zhou et al., 2015, 2017; Wang et al., 2019b), and the results showed that the MMSE score was significantly reduced in the combined treatment group. Three SRs/MAs (Zhou et al., 2015, 2017; Wang et al., 2019b) used ADL scores to evaluate the efficacy of acupuncture plus CT vs. CT in the treatment of AD, and the results showed that there was a significant decrease in ADL scores in the combined treatment group. Furthermore, one review (Wang et al., 2019b) reported that acupuncture plus medication was superior to medication alone for the ADAS-cog score.

## Discussion

SRs/MAs are considered the gold standard for assessing the effects of healthcare interventions, but their methodology must strictly comply with a series of guidelines to minimize the possibility of bias in answering a specific research question. That is, a high quality of SRs/MAs is crucial to ensure the validity, clarity, and accurate comprehension of evidence (Jadad et al., 1998; Balshem et al., 2011). In recent years, the number of SRs/MAs targeting the same topic has been increasing, but their quality is uneven, and their results are not always fully consistent. From the perspective of evidence-based medicine, this phenomenon might impair policy-making and healthcare decisions. Based on the above issues, the research methods of an overview of SR/MA has been proposed by experts in evidence-based medicine (Hunt et al., 2018). An SR/MA overview is a comprehensive research method for re-evaluating a comprehensive collection of SRs/MAs related to the same disease or health problem, and it enables more comprehensively integrating evidence, thus providing higher-quality evidence for clinicians. A literature search revealed that numerous SRs/MAs have been performed to clarify the efficacy and safety of acupuncture in the treatment of AD. However, their quality varied, and the results of these SRs/MAs have limitations. We conducted this systematic overview to synthesize the outcomes from multiple SRs/MAs and to evaluate their quality and attempted to address any inconsistent outcomes. To our knowledge, this overview was the first study to comprehensively evaluate SRs/MAs of acupuncture for AD, and some pivotal findings were found.

### Summary of Main Findings

First, from this overview, we found that the methodological quality, reporting quality, risk of bias, and evidence quality of the included SRs/MAs were unsatisfactory. In AMSTAR-2, all included SRs/MAs were regarded as critically low quality, especially in items 2 (protocol registration), 4 (literature search strategy), 7 (literature screening), 13 (account for RoB), and 15 (publication bias). For reporting quality, inadequate reporting items focused on protocol registration, risk of bias, search, additional analyses, and risk of publication bias. For ROBIS, almost all included SRs/MAs were rated as high risk in phase 3, which results in a consequent decrease in the transparency of SRs/MAs and a consequent increase in the risk of bias. For GRADE, no high-quality evidence was found, and the risk of bias was the most common among the downgrading factors in the included SRs/MAs, followed by imprecision, inconsistency, publication bias, and indirectness. The assessment results of the above tools for the included SRs/MAs from different perspectives revealed common areas for improvement. First, almost all SRs/MAs did not register a protocol, which may result in a larger adjustment of the study process than expected, increasing the risk of bias and affecting the rigor of the systematic review. Second, 8 of 11 SRs/MAs provided only search keywords but no specific search strategy, likely contributing to making the comprehensiveness of the literature search difficult to ensure. Third, all included SRs/MAs did not provide a list of excluded trials with reasons for exclusion, which may undermine the transparency of the SRs/MAs and affect the reliability of their results. Furthermore, the included SRs/MAs have different degrees of shortcomings in the reasonable explanation of bias risk, the data synthesis process, publication bias, and funding support information, which affect the quality of SRs/MAs and reduce the utility of the evidence.

Second, no definitive conclusions can be drawn, and caution is required when acupuncture is recommended as an alternative treatment for AD based on the published results. Among the included SRs/MAs, 8 of 11 reached positive results on acupuncture for AD, and the remaining came to a negative conclusion. Though the research topics of the included SRs/MAs were consistent, all were about acupuncture for AD, and they drew upon the same pool of articles. However, the research conclusions of these SRs/MAs were not consistent. Possible reasons for the inconsistency in conclusions are as follows. First, there was the same article pool among different SRs/MAs on the same topic, and when the number of articles in the article pool accounts for the majority of articles included in all of the SRs/MAs, they are more likely to draw consistent conclusions. We conducted an extraction analysis of all original RCTs included in the SRs/MAs of acupuncture for AD. It was found that 11 SRs/MAs included a total of 137 articles, of which 97 (70.8%) articles appeared only once in all of the SRs/MAs, which means that these 97 articles were not included in the common article pool. The same article pool contained 40 (29.2%) articles. Of these 40 articles, the same article was included from one to five times in the SRs/MAs, including 16 articles that were repeatedly included once, 6 articles that were repeatedly included twice, and 3 articles that were repeatedly included four times. This finding suggests that although the included SRs/MAs are all about acupuncture treatment of AD, their number of articles drawn from the same article pool is too small, and the overall differences among the included studies were large, so this may be one of the reasons why they came to inconsistent conclusions. Second, for GRADE, a risk of bias was the most common (29/33, 87.9%) downgrading factors in the included SRs/MAs, which means that the original trials included in the SRs/MAs were of poor quality. Assessing the methodological quality of the original RCTs, most of them refer only to randomization and do not provide a random sequence generation method; most of the RCTs do not explicitly state that treatment allocation was concealed; only a few RCTs mentioned blinding, and most of the subjects and doctors were not blinded. Well-designed and implemented RCTs are considered the gold standard for evaluating interventions to minimize or avoid bias (Moher et al., 2010). Therefore, when the quality of the included RCTs is unsatisfactory, the risk of bias increases and may ultimately affect the authenticity of the results of SRs/MAs. Furthermore, it is worth noting that although most of the included SRs/MAs indicated that acupuncture appears to be an effective treatment for AD, most authors did not wish to draw definitive conclusions due to the small sample size of the included trials or their low quality. Therefore, more high-quality RCTs with large sample sizes are essential to determine whether acupuncture is beneficial for AD.

Third, all of the SRs/MAs included in this overview were conducted in two Asian countries (10 from China and one from Korea), and no unpublished studies using patients of different races were found, which may lead to a risk of publication bias. The included SRs/MAs were published in both Chinese and English languages, and the articles published in both languages contained negative and positive results. No significant risk of publication bias was found in the Chinese and English language publication forms. Acupuncture is currently used to relieve AD symptoms in many clinics in the West as well as the East, but there has been little research on its effectiveness; thus, this may affect the application of the results for an international population. Further studies on this topic should be carried out in both the East and the West in the future.

### Implications for Future Research

Assessment of various aspects of the included SRs/MAs using the AMSTAR-2, PRISMA, and ROBIS assessments identified areas for common improvement. For example, the reviewer should register or publish the study protocol in advance to avoid any risk of bias and to ensure the rigor of the SR/MA process. In terms of the literature search and selection, the gray literature should be taken into account, and a list of excluded literature with explanations should be provided to guarantee transparency and to avoid publication bias. When conducting data analyses, if the heterogeneity is significant, subgroup analysis or meta-regression should be performed. Funding sources should be mentioned in the reviews because the results of business-funded studies might be biased toward the funder. Researchers should follow the relevant norms of the AMSTAR-2, PRISMA, and ROBIS assessments as much as possible to minimize the possibility of bias in answering a specific research question and to further improve the study quality. For GRADE, future RCTs should address the methodological issues through rigorous trial designs, reasonable appraisals, and critical analyses, and researchers should follow the basic guidelines for reporting clinical trials, such as the CONSORT statement and the STRICTA recommendations. Moreover, studies on this topic should be carried out in both the East and the West in the future.

### Strength and Limitations

As an overview of acupuncture for AD, this study can provide a comprehensive evidence reference for clinical practice. Based on the current results, it may be useful for decision-making for AD treatment in the clinic. In addition, the evaluation process through AMSTAR-2, PRISMA, ROBIS, and GRADE revealed obvious limitations in SRs/MAs and RCTs, which may help guide future high-quality studies. However, it is also limited since the evaluation of quality is a subjective process, and different authors may have their own judgment on each factor, so the results may be different from other reviews, although our overview has been evaluated and checked by two independent authors.

## Conclusion

This overview suggests that acupuncture is a promising complementary treatment for AD. However, the low quality of the SRs/MAs supporting these results is of concern. Future studies can be improved by adequately reporting the methodological details and adhering to the guidelines for conducting such reviews. The clinical effectiveness of acupuncture for AD should be tested in future RCTs with larger sample sizes.

## Author Contributions

JH and MS planned and designed the study. MW, SL, and XQ screened potential studies and extracted data from the included studies. JH and MS assessed the quality and summarized the evidence. JH wrote the first draft. YH revised the draft. All authors approved the final version of the manuscript.

## Conflict of Interest

The authors declare that the research was conducted in the absence of any commercial or financial relationships that could be construed as a potential conflict of interest.

## Abbreviations

AD: Alzheimer's disease
SR: systematic review
MA: meta-analysis
AMSTAR-2: Assessing the Methodological Quality of Systematic Reviews 2
ROBIS: Risk of Bias in Systematic Reviews
PRISMA: Preferred Reporting Items for Systematic Reviews and Meta-Analyses
GRADE: Grading of Recommendations, Assessment, Development, and Evaluation
FDA: Food and Drug Administration
RCTs: randomized clinical trials
CT: conventional therapy
CNKI: China National Knowledge Infrastructure
MMSE: Mini-Mental State Examination Score
ADL: Activities of Daily Living Scale
ADAS-cog: Alzheimer's Disease Assessment Scale-Cognition
HDS: Hasegawa's Dementia Scale
PICO: Population, Intervention, Control Group, and Outcome.
