# Supplementary material for: Acupuncture for the Treatment of Alzheimer's Disease: An Overview of Systematic Reviews
Source: Front Aging Neurosci. 2020 Nov 27;12:574023. doi: 10.3389/fnagi.2020.574023 (PMC7729156; doi:10.3389/fnagi.2020.574023)
Supplement: Supplementary file 1 [file Table_1.DOCX]

**Appendix 1. Search strategies of each database**

The following database will be searched from inception to 24, February, 2020.

**Database 1 China National Knowledge Infrastructure (CNKI)**

SU=('阿尔茨海默病' +'老年性痴呆'+'呆症') AND SU=('针刺'+'针灸'+'耳针'+'体针'+'舌针'+'电针'+'温针灸'+'腹针'+'头针') AND SU=('荟萃分析'+'系统综述'+'系统评价'+'Meta分析')

**Database 2 Wanfang Database**

(阿尔茨海默病 or 老年性痴呆 or 呆症) and (针刺 or 针灸or耳针or体针or舌针or电针or温针灸or腹针or头针) and (荟萃分析 or 系统综述 or 系统评价 or Meta分析)

**Database 3 Chongqing VIP**

(U=阿尔茨海默病 OR U=老年性痴呆 OR U=呆症) AND (U=针刺 OR U=针灸 OR U=耳针 OR U=体针 OR U=舌针 OR U=电针 OR U=温针灸 OR U=腹针 OR U=头针) AND (U=荟萃分析 OR U=系统综述 OR U=系统评价 OR U=Meta分析)

**Database 4 Web of science**

Web of science：TS=(‘Alzheimer Disease’ OR ‘Alzheimer's Disease’ OR ‘Alzheimer Dementia’ OR ‘Alzheimer*’ OR ‘Alzheimer Type Dementia’ OR ‘Senile Dementia’ OR ‘AD’ OR ‘ATD’) AND TS=(‘Acupuncture’ OR ‘Pharmacoacupuncture’ OR ‘Acupotomy’ OR ‘Acupotomies’ OR ‘Pharmacopuncture’ OR ‘needle’ OR ‘needling’ OR ‘dry-needling’ OR ‘body-acupuncture’ OR ‘electroacupuncture’ OR ‘electro-acupuncture’ OR ‘auricular acupuncture’ OR ‘warm needle) AND TS=(‘systematic review*’ OR ‘meta-analysis’ OR ‘meta-analyses’ OR ‘meta analysis’)

**Database 5 Sino-Med**

1 "阿尔茨海默病" [不加权:扩展]

2 "阿尔茨海默病"[常用字段:智能] OR "老年性痴呆"[常用字段:智能] OR "呆症"[常用字段:智能]

3 1 OR 2

4 "针刺疗法" OR "针刺" OR "针灸疗法" [不加权:扩展]

5 "针刺"[常用字段:智能] OR "针灸"[常用字段:智能] OR "耳针"[常用字段:智能] OR "电针"[常用字段:智能] OR "体针"[常用字段:智能] OR "舌针"[常用字段:智能] OR "温针灸"[常用字段:智能] OR "头针"[常用字段:智能] OR "腹针"[常用字段:智能]

6 4 OR 5

7 "Meta分析"[不加权:扩展]

8 "Meta分析"[常用字段:智能] OR "系统评价"[常用字段:智能] OR "荟萃分析"[常用字段:智能] OR "系统综述"[常用字段:智能]

9 7 OR 8

10 3 AND 6 AND 9

**Database 6 Pubmed**

1 Alzheimer Disease [Mesh]

2 Alzheimer Disease [Title/Abstract] OR Alzheimer's Disease [Title/Abstract] OR Alzheimer Dementia [Title/Abstract] OR Alzheimer* [Title/Abstract] OR Alzheimer Type Dementia [Title/Abstract] OR Senile Dementia [Title/Abstract] OR AD [Title/Abstract] OR ATD [Title/Abstract]

3 1 OR 2

4 Acupuncture[Mesh]

5 Acupuncture[Title/Abstract] OR acupotomy[Title/Abstract] OR acupotomies[Title/Abstract] OR needle[Title/Abstract] OR needling[Title/Abstract] OR dry-needling[Title/Abstract] OR body-acupuncture[Title/Abstract] OR electroacupuncture[Title/Abstract] OR electro-acupuncture[Title/Abstract] OR auricular acupuncture[Title/Abstract] OR warm needle[Title/Abstract] OR pharmacoacupuncture[Title/Abstract] OR pharmacopuncture[Title/Abstract]

6 4 OR 5

7 Meta-Analysis as Topic[Mesh]

8 Systematic review[Title/Abstract] OR Meta-Analysis[Title/Abstract] OR meta-analyses[Title/Abstract] OR metaanalysis[Title/Abstract]

9 7 OR 8

13 3 AND 6 AND 9

• # 1 Mesh descriptor: [Alzheimer Disease] explode all trees;

# 2 "Alzheimer Disease":ti,ab,kw or "Alzheimer's Disease":ti,ab,kw or "Alzheimer Dementia":ti,ab,kw or " Alzheimer*":ti,ab,kw or "Alzheimer Type Dementia":ti,ab,kw or "Senile Dementia":ti,ab,kw or " AD":ti,ab,kw or "ATD":ti,ab,kw (Word variations have been searched);

•#3 #1 or #2

•#4 Mesh descriptor: [Acupuncture] explode all trees;

•#5 "acupuncture":ti,ab,kw or "pharmacoacupuncture":ti,ab,kw or "acupotomy":ti,ab,kw or "acupotomies":ti,ab,kw or "pharmacopuncture":ti,ab,kw or "needle":ti,ab,kw or "needling":ti,ab,kw or "dry-needling":ti,ab,kw or "body-acupuncture":ti,ab,kw or "electroacupuncture":ti,ab,kw or "electro-acupuncture":ti,ab,kw or "auricular acupuncture":ti,ab,kw or "warm needle":ti,ab,kw (Word variations have been searched);

•#6 #4 or #5;

•#7 Mesh descriptor: [Meta-Analysis as Topic] explode all trees;

•#8 Mesh descriptor: [Meta-Analysis] explode all trees;

•#9 "systematic review":ti,ab,kw or "Meta-Analysis":ti,ab,kw or "meta analysis":ti,ab,kw or "meta-analyses":ti,ab,kw or "metaanalysis":ti,ab,kw (Word variations have been searched);

•#10 #7 or #8 or #9

•#11 #3 and #6 and #10

**Database 7 Cochrane Library**

1 Mesh descriptor: [Alzheimer Disease]explode all trees;

2 Alzheimer Disease:ti,ab,kw or Alzheimer's Disease:ti,ab,kw or Alzheimer Dementia:ti,ab,kw or Alzheimer*:ti,ab,kw or Alzheimer Type Dementia:ti,ab,kw or Senile Dementia:ti,ab,kw or AD:ti,ab,kw or ATD:ti,ab,kw (Word variations have been searched)

3 1 or 2

4 Mesh descriptor: [Acupuncture] explode all trees;

5 acupuncture:ti,ab,kw or acupotomy:ti,ab,kw or acupotomies:ti,ab,kw or needle:ti,ab,kw or needling:ti,ab,kw or "dry- needling:ti,ab,kw or body-acupuncture:ti,ab,kw or electroacupuncture:ti,ab,kw or electro-acupuncture:ti,ab,kw or auricular acupuncture:ti,ab,kw or warm needle:ti,ab,kw or pharmacoacupuncture:ti,ab,kw or pharmacopuncture:ti,ab,kw (Word variations have been searched);

6 7 or 8

9 Mesh descriptor: [Meta-Analysis as Topic] or [Meta-Analysis]explode all trees;

10 systematic review:ti,ab,kw or Meta-Analysis:ti,ab,kw or meta analysis:ti,ab,kw or meta-analyses:ti,ab,kw (Word variations have been searched);

11 9 or 10

12 3 and 6 and 9

**Database 8 EMBASE**

1 'Alzheimer Disease'/exp

2 'Alzheimer Disease':ab,ti OR 'Alzheimer Dementia':ab,ti OR ' Alzheimer*':ab,ti OR 'Alzheimer Type Dementia':ab,ti OR 'Senile Dementia':ab,ti OR 'AD':ab,ti OR 'ATD':ab,ti

3 1 OR 2

4 'acupuncture'/exp

5 'acupuncture':ab,ti OR 'acupotomy':ab,ti OR 'acupotomies':ab,ti OR 'needle':ab,ti OR 'needling':ab,ti OR 'dry-needling':ab,ti OR 'body-acupuncture':ab,ti OR 'electroacupuncture':ab,ti OR 'electro-acupuncture':ab,ti OR 'auricular acupuncture':ab,ti OR 'warm needle':ab,ti OR 'pharmacoacupuncture':ab,ti OR 'pharmacopuncture':ab,ti

6 4 OR 5

7 'meta analysis'/exp OR 'meta analysis (topic) '/exp OR 'systematic review'/exp OR 'systematic review (topic) '/exp

8 'systematic review':ab,ti OR 'Meta-Analysis':ab,ti OR 'meta analysis':ab,ti OR 'meta-analyses':ab,ti OR 'metaanalysis':ab,ti

9 7 or 8

10 3AND 6 AND 9

**Appendix 2.** **Full text articles excluded with reasons**

| **Full text articles excluded** | **Reasons** |
| --- | --- |
| Li, 2019 | The intervention does not meet the criteria |
| Lin, 2018 | Graduate dissertation without peer review |
| Cui, 2014 | Not rigorous SR/MA done by only one author |
| Mason, 2013 | The subjects n does not meet the criteria |
| Tian, 2012 | Not providing sufficient information |

Li J and Liu JJ. Meta-analysis on the therapeutic effect of acupuncture combined with medicine on Alzheimer's disease. Chinese Journal of Alzheimer’s Disease. 0(3), 2019.

Lin FC. A Systematic Review and Meta-analysis of Acupuncture for Alzheimer's Disease. Guangzhou University of Chinese Medicine. 2018.

Cui Y. Meta-analysis of Acupuncture for Alzheimer's Disease. Inner Mongolia Journal of traditional Chinese Medicine. 33(23), 2014.

Mason CPL, Ka KY, Chung T, et al. Acupuncture improves cognitive function: A systematic review.

Neural Regeneration Research. 8(18), 1673-1684, 2013.

Tian TT, Zhang YL, Cui YW, ET AL. A Systematic Review of Acupuncture Versus Western Medicine for Alzheimer's Disease. Journal of changchun university of traditional chinese medicine. 28(1), 2012.
